# Jasmonic Acid-Induced β-Cyclocitral Confers Resistance to Bacterial Blight and Negatively Affects Abscisic Acid Biosynthesis in Rice

**DOI:** 10.3390/ijms24021704

**Published:** 2023-01-15

**Authors:** Shiduku Taniguchi, Aya Takeda, Masaki Kiryu, Kenji Gomi

**Affiliations:** Faculty of Agriculture, Kagawa University, Miki 761-0795, Kagawa, Japan

**Keywords:** abscisic acid, jasmonate, plant volatile, rice, *Xanthomonas oryzae* pv. *oryzae*

## Abstract

Jasmonic acid (JA) regulates the production of several plant volatiles that are involved in plant defense mechanisms. In this study, we report that the JA-responsive volatile apocarotenoid, β-cyclocitral (β-cyc), negatively affects abscisic acid (ABA) biosynthesis and induces a defense response against *Xanthomonas oryzae* pv. *oryzae* (*Xoo*), which causes bacterial blight in rice (*Oryza sativa* L.). JA-induced accumulation of β-cyc was regulated by OsJAZ8, a repressor of JA signaling in rice. Treatment with β-cyc induced resistance against *Xoo* and upregulated the expression of defense-related genes in rice. Conversely, the expression of ABA-responsive genes, including ABA-biosynthesis genes, was downregulated by JA and β-cyc treatment, resulting in a decrease in ABA levels in rice. β-cyc did not inhibit the ABA-dependent interactions between OsPYL/RCAR5 and OsPP2C49 in yeast cells. Furthermore, we revealed that JA-responsive rice carotenoid cleavage dioxygenase 4b (OsCCD4b) was localized in the chloroplast and produced β-cyc both in vitro and in planta. These results suggest that β-cyc plays an important role in the JA-mediated resistance against *Xoo* in rice.

## 1. Introduction

Rice (*Oryza sativa* L.) is an important crop worldwide and has a precise regulatory network that responds to many environmental stresses, including pathogen attack. In rice plants, a few plant hormones induce the expression of numerous defense-related genes, under pathogen attack. The plant hormone, jasmonic acid (JA), plays an important role in the regulation of defense responses in rice [1]. JA induces disease resistance against the hemibiotrophic pathogen, *Xanthomonas oryzae* pv. *oryzae* (*Xoo*), which causes rice bacterial blight [2]. In contrast, the expression of JA-responsive genes is downregulated by virulent *Xoo* infection in rice [3]. A study revealed that treatment with cell-wall-degrading enzymes derived from *Xoo* activates JA signaling and induces *Xoo* resistance in rice [4]. JA upregulates the expression of defense-related genes by activating transcription factors (TFs), such as OsMYC2, OsbHLH034, and RICE EARLY RESPONSIVE TO JASMONATE1 (RERJ1) [5,6,7]. OsMYC2 regulates the expression of early JA-responsive defense-related genes in rice [5]. OsMYC2 and OsbHLH034 interact with several rice JA repressors, JASMONATE ZIM (JAZ) proteins, which forms a complex with NOVEL INTERACTOR OF JAZ (NINJA) [5,6,8]. The degradation of JAZ proteins triggers the release of TFs that regulate the expression of genes involved in JA signaling [9,10]. Transgenic rice plants overexpressing the C-terminal-deleted *OsJAZ8ΔC* exhibit a JA-insensitive phenotype and decreased JA-induced resistance against *Xoo* [10]. Moreover, overexpression of *OsNINJA1* and its interactors, *OsSRO1a* and *OsFHA1*, results in the suppression of OsMYC2-dependent defense responses in rice [8,11,12]. Additionally, a WRKY-type TF, OsWRKY72, directly binds to the promoter of the rice JA biosynthesis gene, *OsAOS1*, and represses its transcription, resulting in the reduction of endogenous JA levels and an increase in *Xoo* susceptibility in rice [13]. In rice, lipase-like protein, OsEDS1, acts as a positive regulator in the JA-mediated defense response against *Xoo*, whereas in *Arabidopsis*, AtEDS1 regulates the salicylic acid (SA) signaling pathway in response to biotrophic pathogen infection [14].

*Xoo* is a vascular pathogen that colonizes the xylem vessels [15] and infects living cells through the pit membranes that separate the xylem lumen from xylem parenchyma cells. Furthermore, infection with an avirulent *Xoo* strain induces thickening of the xylem secondary walls and reduces pit diameter in rice, thus resulting in the reduction of *Xoo* access to xylem parenchyma cells [16]. Lignin is an essential component of the response leading to the thickening of the xylem secondary walls and its accumulation is an important event to protect *Xoo* infection in rice [16,17,18,19]. *Xoo*-derived lipopolysaccharides induce upregulation of lignin biosynthesis genes in rice [20]. OsbHLH034 acts as a positive regulator of lignin biosynthesis for the secondary-wall thickening of xylem vessels in rice [6]. Altogether, these results indicate that JA signaling plays an important role in *Xoo* resistance in rice.

In rice, JA also induces the accumulation of several plant volatiles that are involved in *Xoo* resistance [21,22,23,24]. The C_7_ volatile, (*E,E*)-2,4-heptadienal, has an antibacterial activity against *Xoo* [25]. Additionally, JA-induced volatile terpenoids, such as monoterpenes and sesquiterpenes, exhibit antibacterial activities against *Xoo* [22,23,24]. The monoterpene, γ-terpinene, suppresses *Xoo* growth by damaging the plasma membrane of the *Xoo* cells [22]. Furthermore, geraniol, a monoterpene, inhibits *Xoo* growth by suppressing the expression of cell-division-related genes of *Xoo* [24]. These results suggest that rice possesses several defense mechanisms to suppress *Xoo* infection, and that the JA-responsive volatiles exhibit antibacterial activities against *Xoo* in rice. Furthermore, plant volatiles function as signaling compounds to induce *Xoo* resistance in rice. The C_6_ volatile, (*E*)-2-hexenal, shows antibacterial activity against *Xoo*, and its vapor treatment induces the upregulation of many defense-related genes in rice [26]. Linalool, a JA-responsive monoterpene, also induces disease resistance against *Xoo*, although it has no antibacterial activity against *Xoo* in rice [21,25]. Linalool-accumulating transgenic rice plants, produced by overexpressing *linalool synthase*, exhibited enhanced disease resistance against *Xoo*. Microarray analysis revealed that linalool induces the expression of many defense-related genes in rice [21]. Furthermore, linalool biosynthesis is regulated by OsJAZ8 in rice [21]. RERJ1 interacts with OsMYC2 and positively regulates linalool production by upregulating *linalool synthase* [7]. These results strongly indicate that JA-responsive volatiles play both direct and indirect roles in JA-mediated defense responses in rice.

We previously identified a few JA-responsive volatiles with no antibacterial activity against *Xoo* [21,25]. However, except for linalool, the biological functions of these volatiles in the rice defense response against *Xoo* have not been investigated. In this study, we focused on the JA-responsive volatile, β-cyclocitral (β-cyc), and investigated its role in the JA-mediated defense response against *Xoo* in rice. We demonstrated that β-cyc negatively affects abscisic acid (ABA) biosynthesis. It has been revealed that ABA suppresses SA signaling and has a negative effect on *Xoo* resistance in rice [27]. Furthermore, we showed that in rice, β-cyc is synthesized from β-carotene by the enzymatic reaction of a JA-induced carotenoid cleavage dioxygenase, OsCCD4b.

## 2. Results

### 2.1. JA-Responsive β-Cyc Induces Resistance to Rice Bacterial Blight

**Figure 1:**
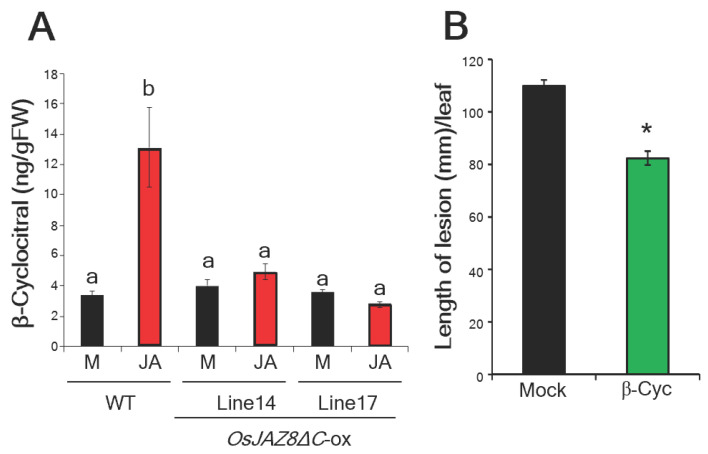
The biosynthesis of JA-inducible beta-cyc is partly dependent on OsJAZ8, and exogenous application of β-cyc increases disease resistance against *Xanthomonas oryzae* pv. *oryzae* (*Xoo*). (**A**) The levels of β-cyc in wildtype (WT) and *OsJAZ8ΔC*-overexpressing plants 24 h after jasmonic acid (JA) treatment (JA). Data were analyzed using the Tukey–Kramer test [*n* = 4 for WT Mock (M); *n* = 6 for WT JA (JA); *n* = 3 for line 14 and line 17 Mock (M); *n* = 4 for line 14 JA; and *n* = 3 for line 17 JA]. Values are expressed as mean ± SE. Means accompanied by different letters are significantly different at *p* < 0.05. (**B**) The length of lesions on β-cyc-treated leaf blades 2 weeks after inoculation with *Xoo*. Values are expressed as means ± SE (*n* = 12). An asterisk represents statistically significant difference from the mock-treated control at *p* < 0.05 (Student’s *t*-test).

Our previous study showed that transgenic rice plants overexpressing *OsJAZ8* with a truncated Jas domain (OsJAZ8ΔC) were insensitive to JA [10]. Large-scale microarray analysis indicated that overexpression of *OsJAZ8ΔC* altered the expression of JA-responsive genes, including defense-related genes, in rice. Furthermore, OsJAZ8ΔC negatively regulates JA-induced resistance to *Xoo* in rice [10]. β-cyc accumulation was not observed in these plants after JA treatment (Figure 1A). This result suggests that the biosynthesis of beta-cyc in rice is induced by JA and is at least partly dependent on OsJAZ8. As shown in Figure 1A, β-cyc is a rice metabolite induced by JA and produced during *Xoo* infection [21]. However, previous research has reported that β-cyc has no antibacterial activity against *Xoo* [25]. Therefore, we decided to investigate the role of β-cyc in *Xoo* resistance in rice. To do this, we applied β-cyc to rice plants and evaluated its effect on disease resistance to *Xoo*. Rice leaf blades were inoculated with virulent *Xoo* 24 h after treatment with β-cyc, and the blight lesion lengths were measured after 2 weeks. As shown in Figure 1B, the blight lesions on the β-cyc-treated rice plants were significantly shorter than those on the mock-treated rice plants. This result indicates that β-cyc has bioactivity in inducing *Xoo* resistance in rice.

### 2.2. β-Cyc Negatively Affects the Expression of ABA-Responsive Genes and ABA Biosynthesis

**Figure 2:**
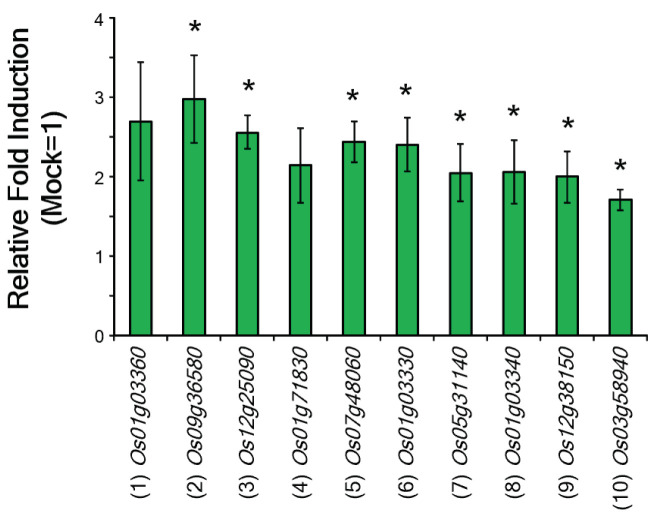
Validation of the microarray results with RT-qPCR. Expression of the selected defense-related genes after β-cyc treatment was analyzed using RT-qPCR. The numbers in parentheses are the same as those in Table 1. Values are expressed as means ± SE (*n* = 4). Asterisks represent statistically significant difference from the mock-treated control at *p* < 0.05 (Student’s *t*-test).

The absence of antibacterial activity of β-cyc against *Xoo* suggests that β-cyc may play a regulatory role in rice defense mechanism(s). Thus, we performed a DNA microarray using the Agilent rice 44 K custom oligo DNA microarray to identify β-cyc-responsive genes in rice. Using the criterion of a two-fold increase or decrease in the average expression levels, we extracted differentially expressed spots in β-cyc-treated rice (Supplementary Table S1). Some of the upregulated genes were associated with the plant defense mechanism, suggesting that they are involved in the β-cyc-induced resistance against *Xoo*. The characteristic defense-related genes are presented in Table 1. To validate the results obtained from microarray analysis, we analyzed the expression of defense-related genes presented in Table 1 using RT–qPCR. The results showed almost the same tendency compared with the microarray results (Figure 2).

**Figure 3:**
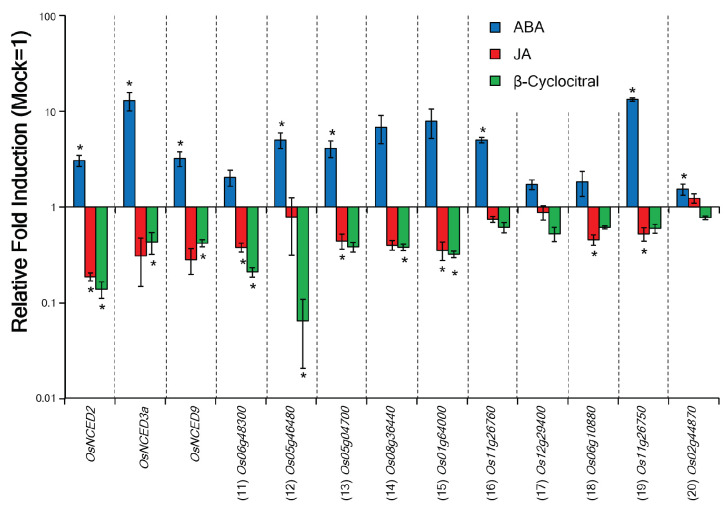
RT-qPCR analysis of abscisic acid (ABA)-responsive genes after ABA, JA, and β-cyc treatment. The numbers in parentheses are the same as those in Table 1. Values are expressed as means ± SE (*n* = 3). Asterisks represent statistically significant difference from the mock-treated control at *p* < 0.05 (Student’s *t*-test).

Analysis of the microarray data revealed that the expression of some ABA-responsive genes was downregulated by β-cyc treatment [28] (Table 1). Thus, we performed RT-qPCR analysis using the ABA-responsive genes and 9-cis carotenoid cleavage dioxygenases (*NCED*s; *OsNCED2*, *OsNCED3a*, and *OsNCED9*), which are involved in ABA biosynthesis [29]. The expression of all the tested genes was confirmed to be upregulated by ABA treatment (Figure 3). Conversely, the expression of all the genes, except for the *dehydrin family protein* [*Os02g44870* (20)], was downregulated by both JA and β-cyc treatments (Figure 3).

**Figure 4:**
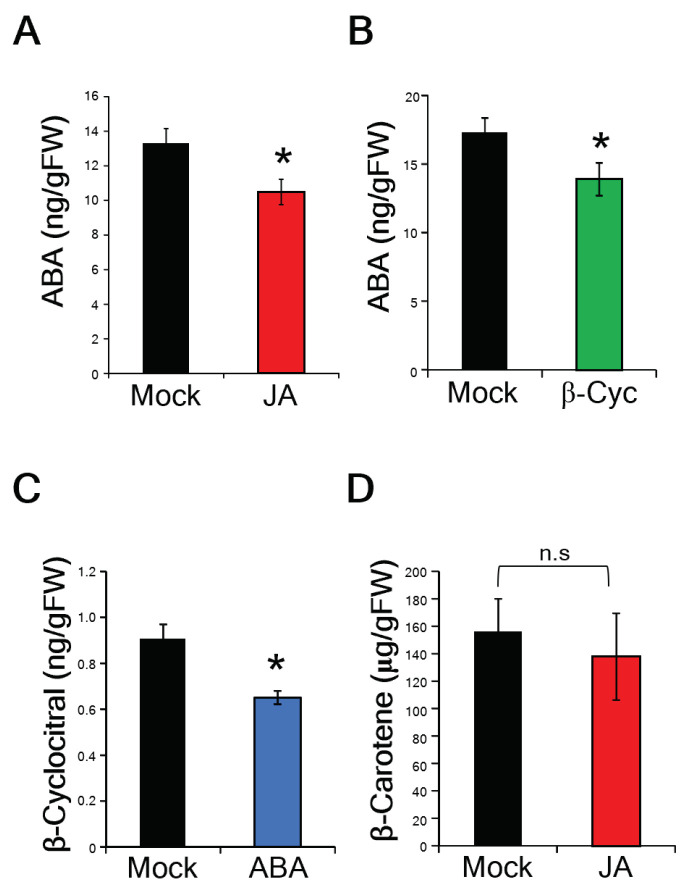
JA and β-cyc negatively affect ABA biosynthesis. (**A**) ABA levels after 24 h of JA treatment. Values are expressed as means ± SE (*n* = 6). (**B**) ABA levels after 24 h of β-cyc treatment. Values are expressed as means ± SE (*n* = 7). (**C**) β-Cyc levels after 24 h of ABA treatment. Values are expressed as means ± SE (*n* = 6). (**D**) β-Carotene levels after 24 h of JA treatment. Values are expressed as means ± SE (*n* = 4). Asterisks represent statistically significant difference from the mock-treated control at *p* < 0.05 (Student’s *t*-test).

Subsequently, we measured the amounts of ABA in the leaf blades post treatments with JA and β-cyc. The levels of ABA in the JA- and β-cyc-treated rice plants were significantly lower than those in mock-treated rice plants (Figure 4A,B). In addition, the levels of β-cyc in the ABA-treated rice plants were significantly lower than those in mock-treated rice plants (Figure 4C). The levels of β-carotene, a precursor for ABA biosynthesis [30], in JA-treated rice plants were not significantly different from those in mock-treated rice plants (Figure 4D).

### 2.3. β-Cyc Negatively Affects ABA-Induced Susceptibility to Xoo

**Figure 5:**
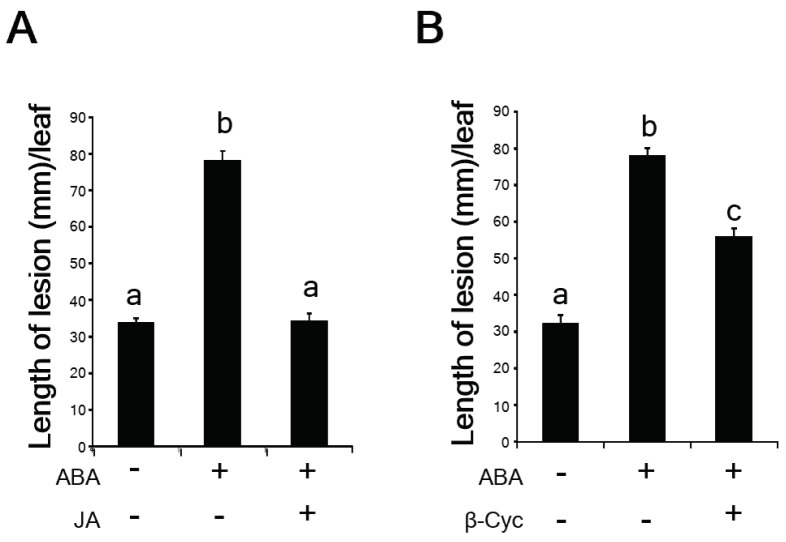
JA and β-cyc negatively affect ABA-induced *Xoo* susceptibility. (**A**) The length of lesions on ABA or ABA/JA-treated leaf blades 8 days after inoculation with *Xoo*. Values are expressed as means ± SE. Data were analyzed using the Tukey–Kramer test [*n* = 12 for Mock (−); *n* = 11 for ABA and ABA/JA (+)]. (**B**) The length of lesions on ABA or ABA/β-cyc-treated leaf blades 8 days after inoculation with *Xoo*. Data were analyzed using the Tukey–Kramer test (*n* = 12 for all treatments). Values are expressed as means ± SE. Means accompanied by different letters are significantly different at *p* < 0.05.

JA induces *Xoo* resistance in rice, while ABA enhances *Xoo* susceptibility [10,27]. When rice plants were inoculated with virulent *Xoo* after treatment with ABA for 24 h, the blight lesions were significantly longer than those on the mock-treated rice plants (Figure 5A). However, when the ABA-treated rice plants were subsequently treated with JA or β-cyc for 24 h, the blight lesions in leaf blades were significantly shorter than those of the ABA-treated rice plants (Figure 5A,B).

### 2.4. β-Cyc Has No Effect on ABA Perception on OsPYL/RCAR5

**Figure 6:**
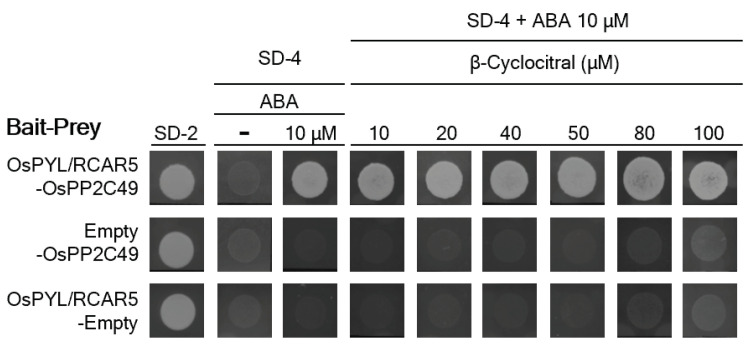
β-cyc has no effect on the ABA-dependent interaction between OsPYL/RCAR5 and OsPPC2C49. The selection media (SD−4) were supplemented with 10 μM ABA and different concentrations of β-cyc.

ABA binds to pyrabactin-resistant like/regulatory components of ABA receptors 5, OsPYL/RCAR5, which forms a complex with a protein phosphatase, OsPP2C49, to regulate ABA signaling in rice [31]. The ABA-dependent interaction between OsPYL/RCAR5 and OsPP2C49 was demonstrated using a yeast two-hybrid system (Y2H) [31]. We added β-cyc to the Y2H medium to investigate whether β-cyc interferes with the ABA-dependent OsPYL/RCAR5-OsPP2C49 interaction, and the result revealed that there was no effect on the ABA-dependent OsPYL/RCAR5-OsPP2C49 interaction at any concentration of β-cyc (Figure 6).

### 2.5. Identification of a Rice β-Cyc Biosynthesis Gene, OsCCD4b

**Figure 7:**
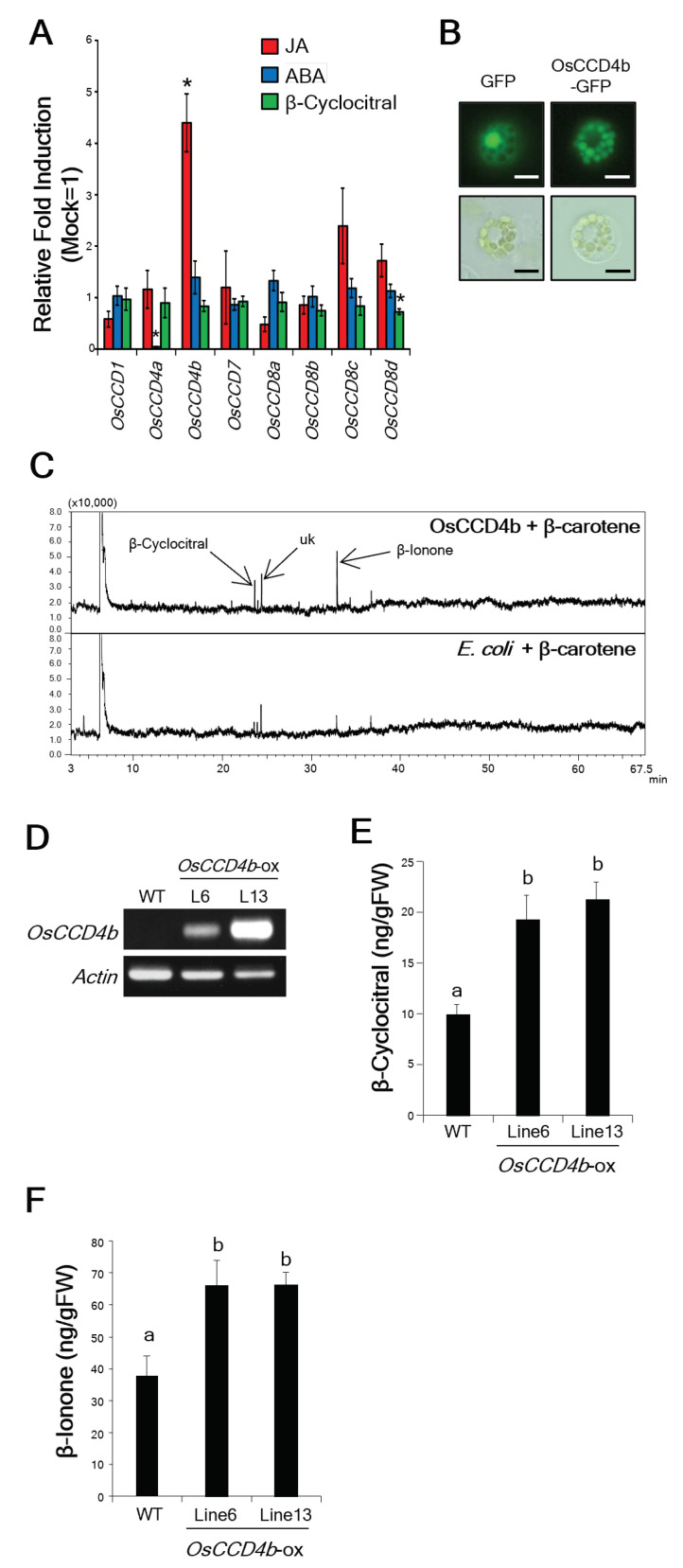
Identification of β-cyc biosynthesis gene, OsCCD4b. (**A**) RT-qPCR analysis of rice *CCD* family genes after treatments with JA, ABA, and β-cyc. Values are expressed as means ± SE (*n* = 4). Asterisks represent statistically significant difference from the mock-treated control at *p* < 0.05 (Student’s *t*-test). (**B**) Subcellular localization of OsCCD4b in rice cells. OsCCD4b-GFP and GFP-only vectors were transferred into rice protoplast cells. Left panels (GFP) show a protoplast expressing the GFP only vector and right panels (OsCCD4b-GFP) show a protoplast expressing the OsCCD4b-GFP vector. Upper panels show fluorescence microscope images and lower panels show light microscope images. Green fluorescence corresponding to the localization of the proteins was detected using fluorescence microscopy. Bars = 10 μm. (**C**) GC profiles of the products catalyzed by His-OsCCD4b protein (upper panel) and by *E. coli* protein (lower panel) using β-carotene as the substrate. Uk: unknown. (**D**) RT-PCR analysis of *OsCCD4b* and *actin* expression in the WT and *OsCCD4b*-overexpressing (*OsCCD4b*-ox) rice plants. (**E**,**F**) β-cyc and β-ionone levels in *OsCCD4b*-overexpressing rice plants. Values are expressed as means ± SE. Data were analyzed using the Tukey–Kramer test (*n* = 7 for WT and line 6; *n* = 8 for line 13). Values are the mean ± SE. Means accompanied by different letters were significantly different at *p* < 0.05.

Recently, it was demonstrated that in saffron (*Crocus sativus*), β-cyc is produced from β-carotene by carotenoid cleavage dioxygenase (CCD)4c, CsCCD4c [32]. The rice genome contains two CCD4 family genes, *OsCCD4a* and *OsCCD4b* [29]. Thus, we investigated the expression of *CCD4* and other CCD family genes after JA, ABA, and β-cyc treatments [29]. The expression of *OsCCD4b* was significantly upregulated by JA treatment (Figure 7A). It has been reported that β-carotene are accumulated in chloroplasts [33]. To investigate localization of OsCCD4b, we constructed an OsCCD4b-GFP fusion gene and transiently expressed the fusion product in rice protoplast cells. This analysis showed that OsCCD4b was localized in the chloroplasts of transformed rice cells (Figure 7B).

To determine the products catalyzed by OsCCD4b, we prepared recombinant histidine-tagged-OsCCD4b (His-OsCCD4b) protein and tested its enzymatic activity using β-carotene as a substrate. The catalyzed products were analyzed using gas chromatography-mass spectrometry (GC-MS). Three major peaks were detected in the His-OsCCD4b reaction with β-carotene (Figure 7C), which were not detected when the total proteins from *E. coli* with an empty vector were analyzed (Figure 7C). The two products were identified as β-cyc and β-ionone by comparing their mass spectra and retention times with those of the authentic β-cyc and β-ionone, respectively. The other product was unknown (Figure 7C).

To assess the enzymatic activity of OsCCD4b in planta, we generated transgenic rice plants overexpressing *OsCCD4b* and confirmed the expression of the transgenes using RT-PCR (Figure 7D). Two independent lines, namely lines six and thirteen, were used for further experiments. The levels of β-cyc and β-ionone in *OsCCD4b*-overexpressing rice plants were significantly higher than those in the wildtype (WT) plants (Figure 7E,F).

## 3. Discussion

JA-induced accumulation of β-cyc was partly regulated by OsJAZ8, suggesting that the activation of the TF that is repressed by OsJAZ8 is essential for JA-dependent β-cyc biosynthesis. OsMYC2 is an OsJAZ8-interacting TF, but microarray analysis of *OsMYC2*-overexpressing rice plants revealed that it does not regulate *OsCCD4b* expression [5]. These results suggest the presence of other uncharacterized TF(s) that play an important role in JA-induced biosynthesis of β-cyc in rice. Although it has no antibacterial activity against *Xoo* in rice [25], treatment with β-cyc induced *Xoo* resistance, suggesting that β-cyc acts as a signaling compound to induce the JA-mediated defense response against *Xoo*. Indeed, in this study, we revealed that the expression of many defense-related genes, including *peroxidase* and *Bowman-Birk type proteinase inhibitor*, was upregulated by β-cyc, which are also upregulated by JA and linalool [10,21]. A β-cyc-responsive peroxidase, OsPrx115, is a secretory-type class III peroxidase involved in lignin biosynthesis [34,35]. Other rice class III peroxidases, OsPrx38 and OsPrx114, play important roles in *Xoo* resistance by producing lignin, which is essential for the thickening of secondary cell walls [6,16]. The expression of *OsPrx38* is regulated by JA-inducible OsbHLH034 [6] and is secreted into the xylem vessels in rice [36]. OsPrx114 is strongly induced by inoculation with avirulent *Xoo* and is secreted into the xylem lumen and walls of xylem parenchyma cells in rice [16]. In addition, it has been reported that transgenic rice plants overexpressing a *Bowman-Birk type proteinase inhibitor* gene exhibited enhanced resistance against *Xoo* [37]. These results suggest that β-cyc-induced resistance is at least partly due to the coordinated expression of these defense-related genes. Further studies, using transgenic rice plants overexpressing *OsPrx115* and β-cyc-responsive *Bowman-Birk proteinase inhibitors* are required to clarify their respective effects on β-cyc-induced *Xoo* resistance in rice.

It has been revealed that ABA has a negative effect on *Xoo* resistance in rice [27]. Rice ABA-deficient mutants exhibit resistance to *Xoo*, and it is thought that the ABA-regulated water potential is involved in *Xoo* resistance [38]. A NAC-type TF, ONAC066, positively regulates *Xoo* resistance by suppressing ABA signaling in rice [39]. Furthermore, ABA has been reported to interact antagonistically with JA and downregulate the expression of the JA biosynthesis gene, *OsAOS2*, and a JA-responsive TF, *OsJAmyb*, in rice [40]. Indeed, ABA treatment suppresses JA production in rice [41]. In this study, we found that exogenous application of β-cyc triggers downregulation of ABA-responsive genes, including ABA-biosynthesis genes, and suppresses ABA-induced susceptibility to *Xoo* in rice. We demonstrated that β-cyc has no effect on the ABA-dependent interaction between OsPYL/RCAR5-OsPP2C49 in yeast cells, suggesting that β-cyc does not inhibit ABA signaling by directly binding to the ABA receptor, OsPYL/RCAR5. The rice genome reportedly contains at least 13 PYL/RCAR and 10 PP2C family genes [31]. Therefore, further analysis of the inhibitory effect of β-cyc on the interactions between other combinations of PYL/RCARs and PP2Cs is required.

Recently, it was reported that β-cyc directly binds to Arabidopsis 1-deoxy-D-xylulose 5-phosphate synthase (AtDXS), a key enzyme in the plastid-localized 2-C-methyl-D-erythritol-4-phosphate (MEP) pathway, and inhibits its enzymatic activity in vitro [42]. The MEP pathway supplies precursors for many metabolites, including carotenoids and their derivatives such as β-carotene and ABA [33]. This finding supports our results, which show a decrease in the ABA levels after JA/β-cyc treatment. However, our previous studies revealed that the production of monoterpenes, such as linalool, γ-terpinene, and geraniol, which are produced via the MEP pathway, is also induced by JA treatment in rice [21,22,24], suggesting that the recognition mechanism of β-cyc in rice is different from that in *Arabidopsis*. Therefore, identification of β-cyc receptors involved in the rice defense mechanism is needed to clarify the biological functions of β-cyc.

It has been reported that ABA also suppresses SA signaling by downregulating *OsWRKY45* and *OsNPR1* expressions [27]. OsWRKY45 was characterized as a key TF involved in SA-mediated disease resistance against *Xoo* [43]. Recently, it has been reported that *OsWRKY45* is a JA-responsive gene and the OsWRKY45-dependent signaling pathway is activated by OsVQ13 in the JA-induced resistance against *Xoo* in rice [44]. OsNPR1 is a rice homologue of an Arabidopsis SA receptor, AtNPR1, and its expression is upregulated by JA [45]. OsNPR1 is degraded by an E3 ubiquitin ligase, OsCUL3a, and *oscul3a* mutant exhibits increased resistance to *Xoo* by activating both JA- and SA-signaling pathways [46]. These results indicate that both JA- and SA-signaling pathways interact coordinately in the rice defense response, termed “Common Defense System” (CDS) [47]. Analysis of the microarray data revealed that neither *OsWRKY45* nor *OsNPR1* were upregulated by β-cyc treatment, suggesting that β-cyc might have no effect on CDS-mediated signaling in rice. However, further studies are required on the involvement of the β-cyc in CDS-mediated defense responses to clarify JA-repressed ABA signaling in rice defense responses.

β-Cyc is a volatile apocarotenoid derived from the oxidative cleavage of β-carotene [48]. The enzymatic cleavage of carotenoids is catalyzed by CCD and NCED family proteins. NCEDs cleave the 11,12 (11′,12′) double bond of 9-*cis*-violaxanthin or 9-*cis*-neoxanthin and are key enzymes in ABA production [49]. CCDs in plants are categorized into four families: CCD1, CCD4, CCD7, and CCD8 [29], among which CCD7 and CCD8 are involved in the biosynthesis of strigolactones, which are plant hormones that regulate plant growth and development [50]. CCD1 has low substrate specificity in vitro and produces several types of apocarotenoids, including β-ionone, along with a wide range of carotenoids [30]. In Arabidopsis, β-cyc and β-ionone are thought to be produced by non-enzymatic oxidative cleavage of β-carotene, but not by CCD-enzymatic cleavage of β-carotene, because these volatiles were normally produced in quadruple mutant for *AtCCD1, 4, 7, and 8* [51]. In contrast, it has been reported that saffron and citrus (*Citrus clementina*) CCD4 proteins, CsCCD4c and CcCCD4b1, respectively, can produce β-cyc in vitro [32,52]. Moreover, CsCCD4c can produce β-ionone [32]. In this study, we demonstrated that OsCCD4b can produce both β-cyc and β-ionone in vitro and in planta. We could not investigate the β-cyc-mediated defense responses in the *OsCCD4b*-ox rice plants, because β-ionone had also accumulated in the plants. We previously found that accumulation of β-ionone was induced by JA treatment in rice [21], suggesting that β-ionone may also be produced by OsCCD4b in JA-treated rice plants. β-Ionone has no antibacterial activity against *Xoo* [25], further studies on β-ionone may provide new insights on the defense responses in rice.

## 4. Materials and Methods

### 4.1. Plant Materials, Chemical Treatments, and Bacterial Inoculation

The growth conditions of the rice plants (*Oryza sativa* L. cv. Hinohikari and Nipponbare) and *Xoo* (strain 7174) were set as previously described by Onohata and Gomi [6]. Nipponbare was used for production of transgenic rice plants. *Xoo* strain is virulent to both Hinohikari and Nipponbare cultivars. Fully opened fifth-leaf blades of rice plants were inoculated using the clipping inoculation technique [53], and the lengths of the blight lesions were measured. *OsJAZ8ΔC*-overexpressing transgenic rice plants produced previously by Yamada et al. [10] were used to analyze the JAZ-dependent regulation of β-cyc. Treatment with 100 μM JA (Sigma, St. Louis, MO, USA), 50 μM ABA (Sigma), and 10 μM β-cyc (Wako, Osaka, Japan) for 24 h was performed as previously described by Taniguchi et al. [21].

### 4.2. Microarray Analysis

An Agilent Rice Oligo Microarray (44 K, custom-made; Agilent Technologies, Redwood City, CA, USA) was used for the microarray analysis. Seedlings of the five-leaf stage rice plants were treated with 10 μM β-cyc for 24 h and a microarray analysis was performed as described by Taniguchi et al. [21]. The microarray data files are stored in the Gene Expression Omnibus Database (accession number GSE152023).

### 4.3. Quantification of Plant Volatiles, ABA, and β-Carotene

The amounts of β-cyc and β-ionone in the leaf blades were measured using GC-MS, as previously described by Kiryu et al. [23]. GC-MS analysis was performed using a GCMS-QP2010SE (Shimadzu, Kyoto, Japan) fitted with a DB-WAX column (60 m × 0.25 mm, 0.25 μm film thickness; J and W Scientific, Folsom, CA, USA) according to the manufacturer’s instructions. The compounds were identified by comparing their mass spectra with those of a database (Wiley10) and their retention times with those of authentic β-cyc and β-ionone. The amount of ABA in the leaf blades was measured using the Phytodetek ABA Test Kit (Agdia, Elkhart, IN, USA), according to the manufacturer’s instructions. β-Carotene was extracted from the leaf blades, as previously described by Zhou et al. [54] and was measured using high-performance liquid chromatography (HPLC). HPLC analysis was performed using a Prominence (Shimadzu, Kyoto, Japan) fitted with an Xbridge C18 column (4.6 mm × 250 mm, 5 μm; Waters, Milford, MA, USA). The extracts were eluted with MeOH-MTBE-H_2_O [81:15:4 (*v*/*v*), solvent A] and MeOH-MTBE-H_2_O [10:90:4 (*v*/*v*), solvent B]. The linier elution gradients started with 100 % of solvent A for 14.5 min at a flow rate of 1 mL min^−1^and were followed by a gradual increase of solvent B, reaching 100% in 2 min, maintaining 100% solvent B for 13.5 min. The peak at 440 nm was identified by comparing the retention time with that of HPLC-grade β-carotene purchased from Sigma-Aldrich (St. Louis, MO, USA).

### 4.4. Reverse Transcription-Quantitative PCR (RT-qPCR)

After each treatment, total RNA was extracted from the rice leaf blades using TRIzol (Invitrogen, Carlsbad, CA, USA), according to the manufacturer’s instructions. Fourth-leaf blades were used for RT-qPCR analysis, and four leaf blades were used per replicate. RT-qPCR was performed as described by Gomi et al. [26]. The sequences of the gene-specific primers used in RT-qPCR are shown in Supplementary Table S2.

### 4.5. Yeast Two-Hybrid (Y2H) System

Y2H analysis was performed as previously described by Kim et al. [31] using the MATCHMAKER Y2H system [Clontech (Takara Bio), Shiga, Japan]. The *OsPYL/RCAR5* (*Os05g12260*) and *OsPP2C49* (*Os05g38290*) cDNAs were ligated into pGBKT7 and pGADT7 vectors, respectively. The plasmids were then introduced into the AH109 yeast strain. The selection media were supplemented with 10 μM ABA and different concentrations of β-cyc. The plates were incubated at 30 °C for 5 days.

### 4.6. Transient Localization Assay

For the construction of *OsCCD4b*-GFP, the ORF of *OsCCD4b* (*Os12g24800*) without the stop codon was amplified by PCR and subcloned into the corresponding site on the pE7133-GFP vector [55], fusing GFP in-frame to the C-terminus of OsCCD4b. The OsCCD4b-GFP fusion protein was introduced into rice protoplast cells using polyethylene glycol 3350, according to Bart et al. [56]. Localization analysis of OsCCD4b was performed as previously described by Kiryu et al. [23]. GFP fluorescence was observed using a BIOREVO BZ-9000 fluorescent microscope (Keyence, Osaka, Japan) equipped with a GFP-specific filter unit.

### 4.7. Functional Expression of OsCCD4b in Escherichia coli

The ORF of *OsCCD4b* truncated the putative chloroplast transit sequence was ligated in-frame into the pCold II vector (Takara, Shiga, Japan) and the histidine-tagged proteins produced in *Escherichia coli* were purified using a HisTrap^TM^ HP column (GE Healthcare, Buckinghamshire, UK) according to the manufacturer’s instructions. The purified fusion protein was dialyzed using an assay buffer [50 mM NaPO_4_ (pH 7.2) containing 300 mM NaCl, 5 μM FeSO_4_, and 5 mM ascorbic acid.]

### 4.8. Enzyme Assay and Analysis of Volatiles

Enzyme activity was assayed using approximately 40 μg of purified His-tagged OsCCD4b protein in a 15 mL sealed Spelco vial (Spelco, St. Louis, MO, USA) containing 1 mL of the assay buffer. The reaction was initiated by the addition of β-carotene (55 μM final concentration). After incubation at 30 °C for 4 h, the headspace above the sample was trapped for 10 min at 50 °C using a polydimethylsiloxane (PDMS)/divinylbenzene (DVB)-coated solid-phase microextraction (SPME) fiber (Supelco, Bellafonte, PA, USA) for GC-MS. The GC-MS conditions were set as described by Kiryu et al. [23].

### 4.9. Production of OsCCD4b-Overexpressing Rice Plants

The ORF of *OsCCD4b* was ligated into the pBI333-EN4 vector [57]. The binary vector was introduced into *Agrobacterium tumefaciens* EHA101 by electroporation [58]. Rice transformation was performed as described by Hiei et al. [59], and the transgenic plants were selected on a medium containing 50 mgL^−1^ of hygromycin. Second and third generation plants were used for the experiments. To verify the expression of the transgene, RT-PCR was performed using the OneStep RT-PCR kit (QIAGEN, Hilden, Germany) with the *OsCCD4b*-specific forward primer and *Nos terminator*-specific reverse primer. The sequences used for RT-PCR are as follows: *OsCCD4bF*, 5’- CAGAGCTAGACATTGTTGCAGAAG-3’ and *tNOSR*, 5’-GTATAATTGCGGGACTCTAATC-3’; *actin*, forward, 5’-CCTGGAATCCATGAGACCAC-3’ and reverse, 5’-ACACCAACAATCCCAAACAGAG-3’.

## Figures and Tables

**Table 1:** List of β-cyclocitral-responsive genes.

		WT + β-Cyc/WT		
Accession Number	Gene Name	Fold Change *	*q*-Value	Reference
**Defense-related gene**				
(1) Os01g03360	Bowman-Birk type bran trypsin inhibitor	8.75	<0.05	This study
(2) Os09g36580	Thaumatin-like protein 1	3.70	<0.05	This study
(3) Os12g25090	Subtilisin/chymotrypsin inhibitor	3.50	<0.05	This study
(4) Os01g71830	Beta-1,3-glucanase	3.44	<0.05	This study
(5) Os07g48060	Peroxidase (OsPrx115)	2.90	<0.05	This study
(6) Os01g03330	Proteinase inhibitor I12	2.72	<0.05	This study
(7) Os05g31140	Beta-glucanase	2.55	<0.05	This study
(8) Os01g03340	Bowman Birk trypsin inhibitor	2.51	<0.05	This study
(9) Os12g38150	Thaumatin	2.25	<0.05	This study
(10) Os03g58940	Lipid transfer protein	2.01	<0.05	This study
**ABA-responsive gene**				
(11) Os06g48300	Protein phosphatase 2C family protein	0.20	<0.05	[28]
(12) Os05g46480	LEA-like protein	0.22	>0.05	[28]
(13) Os05g04700	Similar to ICT protein	0.48	>0.05	[28]
(14) Os08g36440	Abscisic acid and stress inducible (A22) gene	0.50	>0.05	This study
(15) Os01g64000	Similar to ABA response element binding factor	0.54	>0.05	This study
(16) Os11g26760	Dehydrin RAB 16C	0.60	>0.05	[28]
(17) Os12g29400	GRAM domain containing protein	0.71	>0.05	[28]
(18) Os06g10880	Similar to ABA-responsive element binding protein 1	0.71	>0.05	This study
(19) Os11g26750	Dehydrin RAB 16D	0.81	>0.05	[28]
(20) Os02g44870	Dehydrin family protein	0.97	>0.05	[28]

Fold changes (relative to WT plants) and false discovery rate (*q*-values) of defense-related and ABA-responsive genes on β-cyclocitral-treated rice plants from microarray analyses. * Values of fold change are means of 4 independent biological replications. The numbers in parentheses are the same as those in Figure 2 and Figure 3.

## Data Availability

All data generated or analyzed during this study are included in this published article and its supplementary information files. The microarray data files are stored in the Gene Expression Omnibus Database (accession number GSE152023).

## Supplementary Materials

The following supporting information can be downloaded at: https://www.mdpi.com/article/10.3390/ijms24021704/s1.
